# Bevacizumab promotes tenogenic differentiation and maturation of rat tendon-derived cells in vitro

**DOI:** 10.1371/journal.pone.0293463

**Published:** 2023-10-31

**Authors:** Yohei Kusaba, Ken Kumagai, Kimi Ishikawa, Hyonmin Choe, Hiroyuki Ike, Naomi Kobayashi, Yutaka Inaba

**Affiliations:** 1 Department of Orthopaedic Surgery, Graduate School of Medicine, Yokohama City University, Yokohama, Japan; 2 Department of Orthopaedic Surgery, Yokohama City University Medical Center, Yokohama, Japan; Keio University - Shinanomachi Campus: Keio Gijuku Daigaku - Shinanomachi Campus, JAPAN

## Abstract

Previous work suggested that tenogenic differentiation of tendon stem/progenitor cells (TSPCs) was suppressed by upregulated expression of the angiogenic marker vascular endothelial growth factor (VEGF). The purpose of this study was to test the hypothesis that anti-VEGF antibody, bevacizumab, promotes *in vitro* tenogenic differentiation and maturation of two distinct types of TSPCs, tendon proper-derived cells (TDCs), and paratenon-derived cells (PDCs) originating from rat Achilles tendon. TDCs and PDCs were isolated from the tendon proper and the paratenon of rat Achilles tendons. TDCs and PDCs were cultured for 3 days on plates with or without VEGF. TDCs and PDCs were also cultured in collagen gel matrix, and the blocking effect of VEGF was examined by the addition of 100 ng/mL of bevacizumab. Effects of bevacizumab on tenogenic differentiation were assessed using real-time PCR, immunofluorescent staining, and western blotting. VEGF significantly attenuated expression of the *Tnmd* gene in both PDCs and TDCs (*P*<0.05). Expressions of the *Scx*, *Tnmd*, *and Col1a1* genes were significantly upregulated by the addition of bevacizumab (*P*<0.05). Immunofluorescent staining showed that the percentage of tenomodulin-positive PDCs and TDCs was significantly higher with bevacizumab treatment than in control cultures (P<0.05). Western blotting showed that bevacizumab suppressed pVEGFR-2 protein expression in both PDCs and TDCs. Bevacizumab promoted the *in vitro* tenogenic differentiation and maturation of two distinct TSPCs derived from rat Achilles tendon. Since the previous studies demonstrated that TSPCs have a potential to contribute to tendon repair, attenuating VEGF levels in TSPCs by administration of bevacizumab is a novel candidate therapeutic option for promoting tendon repair.

## Introduction

Tendon injuries are difficult to treat, and development of therapeutic strategies remains clinically challenging. Tendon healing is a complex and highly regulated process that involves three overlapping stages: inflammation, proliferation, and remodeling [1]. Multiple molecular factors and cellular elements are involved in these healing processes, and recent efforts to elucidate the underlying functions have been undertaken to devise approaches to augment biological healing.

Tendon stem/progenitor cells (TSPCs) play an important role in cell migration, proliferation, and tenogenic differentiation in the repair of injured tendons [2]. Two distinct stem/progenitor cell populations have been identified, one directly derived from the core of the tendon for intrinsic healing (tendon proper-derived cells; TDCs), and the other derived from the paratenon for extrinsic healing (paratenon-derived cells; PDCs) [3]. These two types of TSPCs respond to the injury site and facilitate tendon repair by expressing tendon-specific markers such as scleraxis [4]. A therapeutic strategy to upregulate the tenogenic induction of TSPCs could augment a patient’s natural healing potential to promote tendon repair.

Growth factors are important mediators in tendon healing processes and control the differentiation of TSPCs [5]. Some research suggests that platelet-rich plasma (PRP), an autologous blood product that contains high concentrations of growth factors, can be used in clinical practice to promote tendon healing [6]. However, the growth factors included in PRP exert both positive and negative effects on the differentiation of TSPCs. A previous study showed that PRP promotes the migration, proliferation, and tenogenic differentiation of TSPCs via upregulation of Scx, but PRP also downregulates the tendon maturation-specific marker tenomodulin (Tnmd) *in vitro* by upregulating expression of the angiogenic marker vascular endothelial growth factor (VEGF) [7].

The present study examined the tenogenic maturation of TSPCs in conjunction with attenuation of VEGF using an anti-VEGF antibody, bevacizumab. The purpose of this study was to test the hypothesis that bevacizumab promotes the *in vitro* differentiation and tenogenic maturation of TSPCs derived from rat Achilles tendon.

## Material and methods

### Animals

The research plan involving rats received the approval of the Animal Research Committee of Yokohama City University (#F-A-15-045). The study involved 12 male Sprague-Dawley rats, aged 6 to 8 weeks and weighing 191 to 301 g (Charles River Laboratories Japan Inc., Kawasaki, Japan). Rats were euthanized by carbon dioxide inhalation after being anesthetized with 2–3 L/min of isoflurane. To avoid causing distress, euthanasia was only carried out after it was confirmed that there was no physical movement under anesthesia, and that consciousness did not return even when stimulated, as previously described [7].

### Isolation of TDCs and PDCs

TDCs and PDCs were harvested from the tendon proper and the paratenon of the Achilles tendon, respectively [8]. The Achilles tendon and paratenon were extracted from the euthanized rats’ ankles. Tendons with attached paratenon were initially incubated for 10 minutes at 37°C using a solution containing 0.5% type I collagenase (CLS-1; Sigma-Aldrich, Darmstadt, Germany) and 0.25% trypsin (Gibco, New York, NY, USA) in high-glucose Dulbecco’s Modified Eagle Medium (DMEM; Wako Pure Chemical, Osaka, Japan). Subsequently, the paratenon was carefully separated from the tendon proper. Residual tendon tissues were sectioned into 1-mm^3^ fragments and further incubated for 20 minutes at 37°C in a solution of 3 mg/mL CLS-1 and 4 mg/mL Dispase II (Wako Pure Chemical). Both the paratenon and tendon proper were subsequently processed through a 70-μm cell strainer. The resulting cells underwent centrifugation at 400 *×g* for 10 minutes, followed by resuspension in growth medium; this medium comprised high-glucose DMEM enriched with 10% fetal bovine serum (FBS; Biowest, Nuaille, France), 100 U/mL penicillin, and 100 μg/mL streptomycin (Gibco). The isolated cells were plated in 6-well plates and maintained under standard conditions of 37°C, 5% CO_2_, and high humidity. Cells derived from the paratenon were called PDCs, whereas those from the tendon proper were called TDCs, as previously described [7].

### 2D cell culture of TSPCs and treatment with VEGF

Third-passage PDCs and TDCs were cultured in 10-cm-diameter dishes at a density of 5.0 × 10^4^ cells/mL. The culture medium used was high-glucose DMEM supplemented with 10% FBS. Cells were maintained under standard conditions at 37°C in an atmosphere of 5% CO_2_ and high humidity for 4 days. The culture medium was changed on the second day, the medium was supplemented with or without VEGF-A (Funakoshi, Tokyo, Japan #NBP3-18190) (10 ng/mL), and the cells were cultured for an additional 3 days.

### 3D cell culture of TSPCs and treatment with bevacizumab

PDCs and TDCs were cultured under 3D conditions to maintain a tenogenic phenotype [7]. The wells of a 12-well plate were coated with 1.5 mL of 2% agarose (Funakoshi, Tokyo, Japan) and formed into an 8 × 15 × 4 mm^3^ semi-cylindrically-shaped mold. Polyethylene terephthalate artificial ligaments (Telos; Aimedic MMT, Tokyo, Japan) were placed on both sides. Four passages of PDCs and TDCs were mixed in type I-A collagen gel matrix (Cellmatrix; Nitta Gelatin, Osaka, Japan) at a density of 1.0 × 10^6^ cells/mL and seeded at 400 μL per mold. PDCs and TDCs were incubated in DMEM containing 10% FBS at 37°C, 5% CO_2_, and high humidity in a controlled environment. The blocking effect of VEGF was examined by the addition of 100 μg/mL of bevacizumab (Avastin, Chugai Pharmaceutical Co., Ltd., Tokyo, Japan). The culture medium was replaced every 2 to 3 days for a duration of 14 days, as previously described [7].

### Real-time reverse transcription polymerase chain reaction

Total RNA was extracted from both PDCs and TDCs on day 7 for 2D cultures and on days 7 and 14 for 3D cultures using RLT Lysis Buffer (Qiagen, Venlo, The Netherlands). RNA concentration was determined by measuring the absorbance at 260 nm, whereas its purity was gauged using the 260/280 nm absorbance ratio. Reverse transcription of 1 μg of total RNA into first-strand cDNA was performed in a 20-μL reaction volume, using the iScript Advanced cDNA Synthesis kit (Bio-Rad, Richmond, CA, USA). Subsequent quantitative real-time PCR analyses were conducted with TaqMan gene expression assays (Applied Biosystems, Waltham, MA, USA) on a CFX96TM real-time PCR detection system (Bio-Rad) in a 20-μL reaction setup. The results of real-time PCR were analyzed using the ΔΔCt method to calculate the relative gene expression levels. Gene expression levels of the target genes under investigation were standardized to the expression of the housekeeping gene, glyceraldehyde-3-phosphate dehydrogenase (GAPDH). The subsequent TaqMan gene expression assays used were as follows: *scleraxis* (*Scx*, Rn01504576_m1), *Tnmd* (Rn00574164_m1), *collagen type 1 alpha 1* (*Col1a1*, Rn01463848_m1), *collagen type 3 alpha 1* (*Col3a1*, Rn01437681_m1), *VEGF-A* (*VEGF*, Rn01511602_m1), and *GAPDH* (Rn01775763_g1).

### Immunofluorescent staining of Tnmd

**Fig 1:**
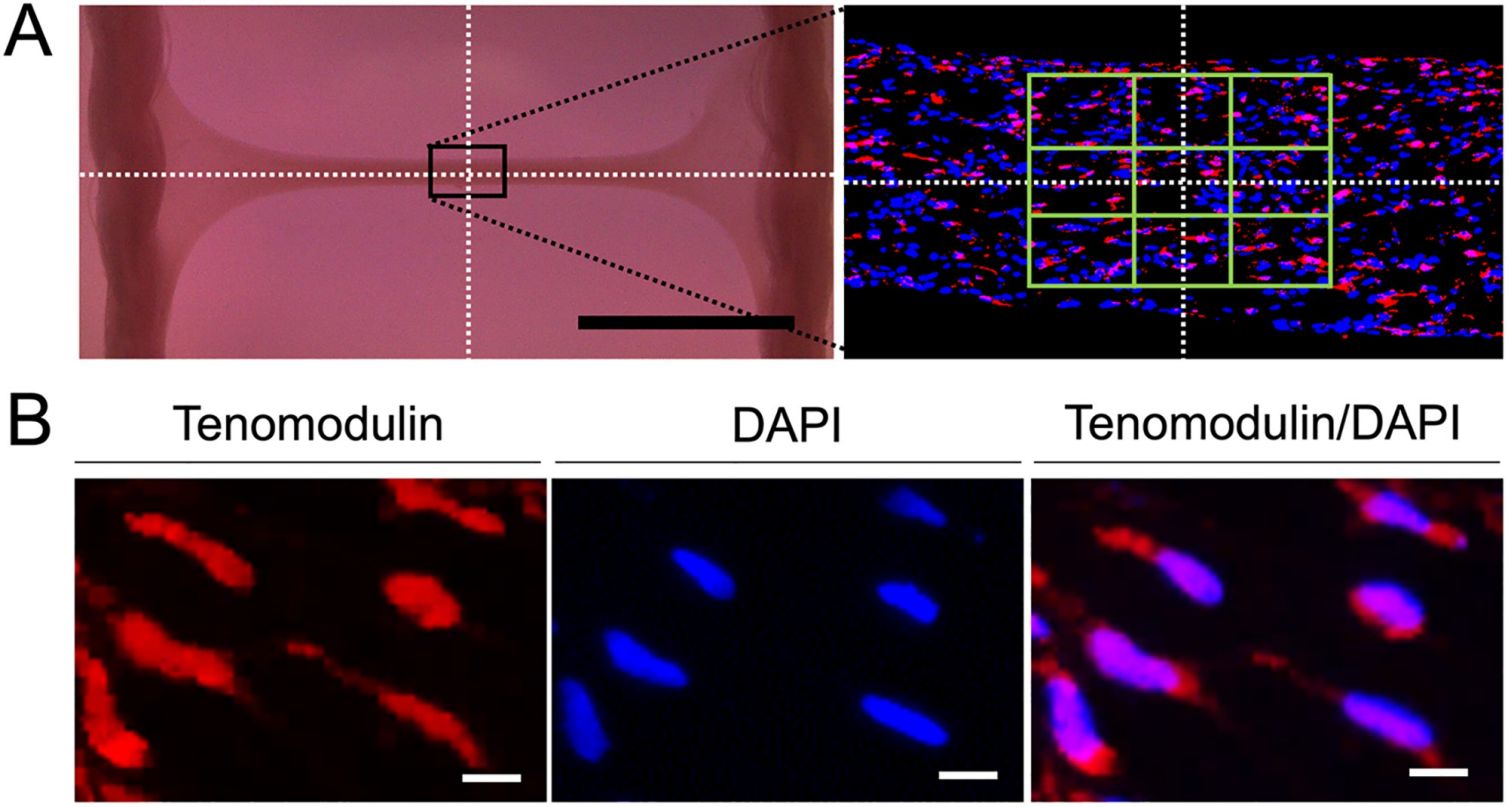
Enumeration of Tnmd-positive cells by immunofluorescence staining. (A) Both PDCs and TDCs develop into tendon-like structures with 14 days of 3D culture. Each of these structures was stained with immunofluorescent stains and nine high-power fields from the central region of each section were captured. Scale bar = 5 mm. (B) Images of Tnmd (red) and DAPI (blue) are overlaid. The number of Tnmd-positive cells relative to DAPI was quantified using BZ-X800 analyzer software. Scale bar = 10 μm.

PDCs and TDCs cultured like tendons on day 14 were washed with phosphate-buffered saline (PBS) and then fixed with 4% paraformaldehyde at 4°C for 24 h. The cells were then embedded in paraffin to prepare sections. The sections were hydrophilized with xylene and ethanol and then treated with L.A.B solution (Liberate Antibody Binding Solution, Polysciences Inc., Warrington, PA, USA) for antigen activation. The sections were then blocked with Blocking One Histo (Nacalai Tesque, Inc., Kyoto, Japan) at room temperature for 1 h and subsequently incubated with anti-rabbit primary antibodies against Tnmd (1:100; LSBio, Seattle, WA, USA) overnight at 4°C. The specificity of the anti-Tnmd antibody was demonstrated by detecting a band at 45 kDa on western blotting (S1 Fig). The sections were then washed three times with 0.1% Tween 20 in PBS and incubated with Alexa Fluor^®^ 568–conjugated goat anti-rabbit IgG secondary antibody (1:500; Invitrogen, Carlsbad, CA, USA) for 45 min at room temperature. To visualize nuclei, the cells were double-stained with 4’,6-diamidino-2-phenylindole (DAPI) (Vector Laboratories, Burlingame, CA, USA). The cells were viewed under an all-in-one fluorescence microscope (BZ-X800, Keyence, Osaka, Japan) equipped with a digital camera (CFI 60, Nikon Corp., Tokyo, Japan). All immunofluorescence images were captured with identical exposure settings. Nine areas encompassing the intersection of the perpendicular bisectors of the tendon-like structures were observed. The number of Tnmd-positive cells among cells stained with DAPI was determined from captured images and expressed as a percentage (Fig 1).

### Western blotting

PDCs and TDCs cultured for 14 days were washed with PBS (pH 7.4) and lysed with sample buffer for western blotting. Equal amounts of protein from each lysate were subjected to 8/16% sodium dodecyl sulfate–polyacrylamide gel electrophoresis (SDS-PAGE) and transferred onto a PVDF membrane (Bio-Rad). The membrane was blocked with Blocking One (Nacalai Tesque) or 5% bovine serum albumin (Nacalai Tesque) in 0.05% Tween 20/Tris-buffered physiological saline at room temperature for 1 h and then incubated overnight at 4°C with anti-β-actin antibody (1:1000; Abcam, Cambridge, UK), anti-VEGF receptor 1 and 2 antibody (1:1000; Abcam), or anti–phospho-VEGFR-1 (Tyr1333) and 2 (Tyr951) antibody (1:500; Affinity Biosciences, Cincinnati, OH, USA). The blots were then incubated with the secondary antibody for 1 h at room temperature. Immunoreactive proteins were detected using ECL Select Western Blotting Detection Reagent (Cytiva, Tokyo, Japan) and exposure to WSE-6100H LuminoGraph (Atto Co., Tokyo, Japan) for a specific time optimal for that antibody.

### Effects of bevacizumab on proliferation, migration, and toxicity

The CCK-8 cell proliferation assay (DOJINDO, Kumamoto, Japan) was used. Passage 5 PDCs and TDCs were seeded at a concentration of 5 × 10^5^ cells/mL, with 100 μL in each well of a 96-well plate. Bevacizumab was administered at concentrations of 10 μg/mL and 100 μg/mL 24 hours later, and the absorbance was measured on days 1, 2, and 3 using a spectrophotometer (S2 Fig).

The scratch assay (Oris cell migration assay, Funakoshi, Tokyo, Japan) was also used. Passage 5 PDCs and TDCs were seeded at a concentration of 5 × 10^5^ cells/mL, with 100 μL in each well of a 96-well plate. After 24 hours, the stopper was removed, and bevacizumab was administered at concentrations of 10 μg/mL and 100 μg/mL. Furthermore, after an additional 24 hours, the samples were stained and analyzed using the Keyence BZ-X800. The percentage of the blue area relative to the area of the circle observed was calculated (S3 Fig).

To assess the cytotoxicity of bevacizumab, apoptosis was evaluated with the CF Dye TUNEL Assay Apoptosis Detection Kits (Biotium, Fremont, CA, USA). Tendon progenitor/stem cells, namely PDCs and TDCs, were allocated to the bevacizumab and control groups and cultured for 14 days. Following this period, the cells were fixed using 4% PFA, after which paraffin-embedded tissue sections were prepared systematically. These sections were then deparaffinized and rehydrated as per established protocols, and they were subsequently subjected to two washes using PBS. To permeabilize the sections, they were treated with a solution of 20 ug/mL proteinase K in PBS and incubated for 30 minutes at 37°C. This was followed by a rinse in PBS and two subsequent 5-minute washes in the same solution. The next phase involved the incubation of the samples in TUNEL Equilibration Buffer for a span of 5 minutes. Once prepared, the Equilibration Buffer was carefully removed, making way for the introduction of 50 uL of the TUNEL reaction mix to every specimen. To ensure optimal cell staining, a diligent incubation period of 1.5 hours at 37 ºC was maintained. Subsequent to the incubation, samples were thrice rinsed for periods of 5 minutes in a specialized PBS solution containing both 0.1% Triton X-100 and 5 mg/mL (0.5%) BSA. For microscopic evaluation, they were sealed in DAPI-containing mounting medium. Measurements were made using the BZ-X800, where apoptosis was stained in red. The ratio of red to DAPI was expressed as a percentage (S4 Fig).

### Statistical analysis

Statistical analysis was carried out using SPSS statistics software (IBM, Tokyo, Japan). Data are represented as mean ± standard error of the mean (SEM) values. Friedman’s test or the Mann-Whitney *U* test was used to examine the significance of differences among the test groups. An adjusted P value of less than 0.05 was deemed significant.

## Results

### Effect of VEGF on the expressions of tenogenic differentiation–related genes in TSPCs

**Fig 2:**
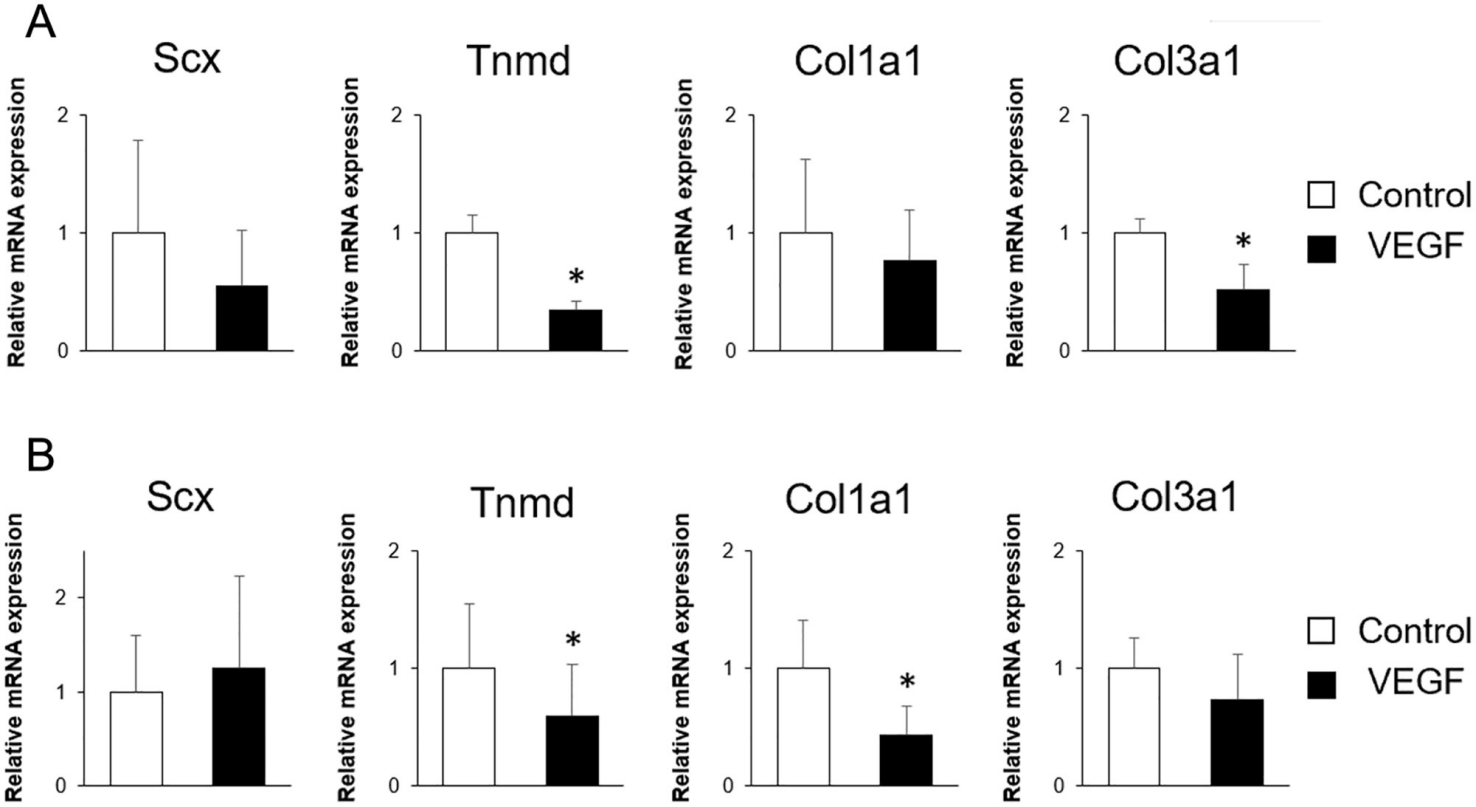
Effects of VEGF on the expression of tenogenic differentiation–related genes in TSPCs. After being cultured under standard conditions for 4 days, PDCs and TDCs were treated with 10 ng/mL of VEGF-A and cultured for an additional 3 days. The relative mRNA expressions of the *Scx*, *Tnmd*, *Col1a1*, and *Col3a1* genes in PDCs (A) and TDCs (B). N = 4, *P<0.05 vs control.

Since a previous study showed that PRP attenuates the expression of Tnmd with upregulation of VEGF in TSPCs [7], the effects of VEGF on the expressions of tenogenic markers such as *Tnmd* in TSPCs were investigated using real-time PCR. In PDCs, the expressions of both *Tnmd* and *Col3a1* were significantly reduced by the addition of VEGF (Fig 2A). In TDCs, *Tnmd* and *Col1a1* expression was significantly reduced by the addition of VEGF (Fig 2B). These results suggest that VEGF suppresses the tenogenic differentiation and maturation of TSPCs.

To determine the appropriate concentration of VEGF, it was administered at concentrations of 1, 10, and 100 ng/mL. *Tnmd* levels decreased significantly at both 10 and 100 ng/mL. Since the expression levels of *Tnmd* were nearly identical at these concentrations, VEGF at 10 ng/mL was selected (S5 Fig).

### Effects of bevacizumab on the expression of tenogenic differentiation-related genes in TSPCs

The initial plan was to conduct bevacizumab administration experiments in 2D culture, similar to the VEGF administration experiments. However, in our preliminary experiments, contrary to our expectations, there were no notable alterations in Tnmd expression (S6 Fig). Therefore, to better manifest the tendon phenotype, experiments were performed by creating a structurally tendon-like construct using 3D culture techniques [8].

**Fig 3:**
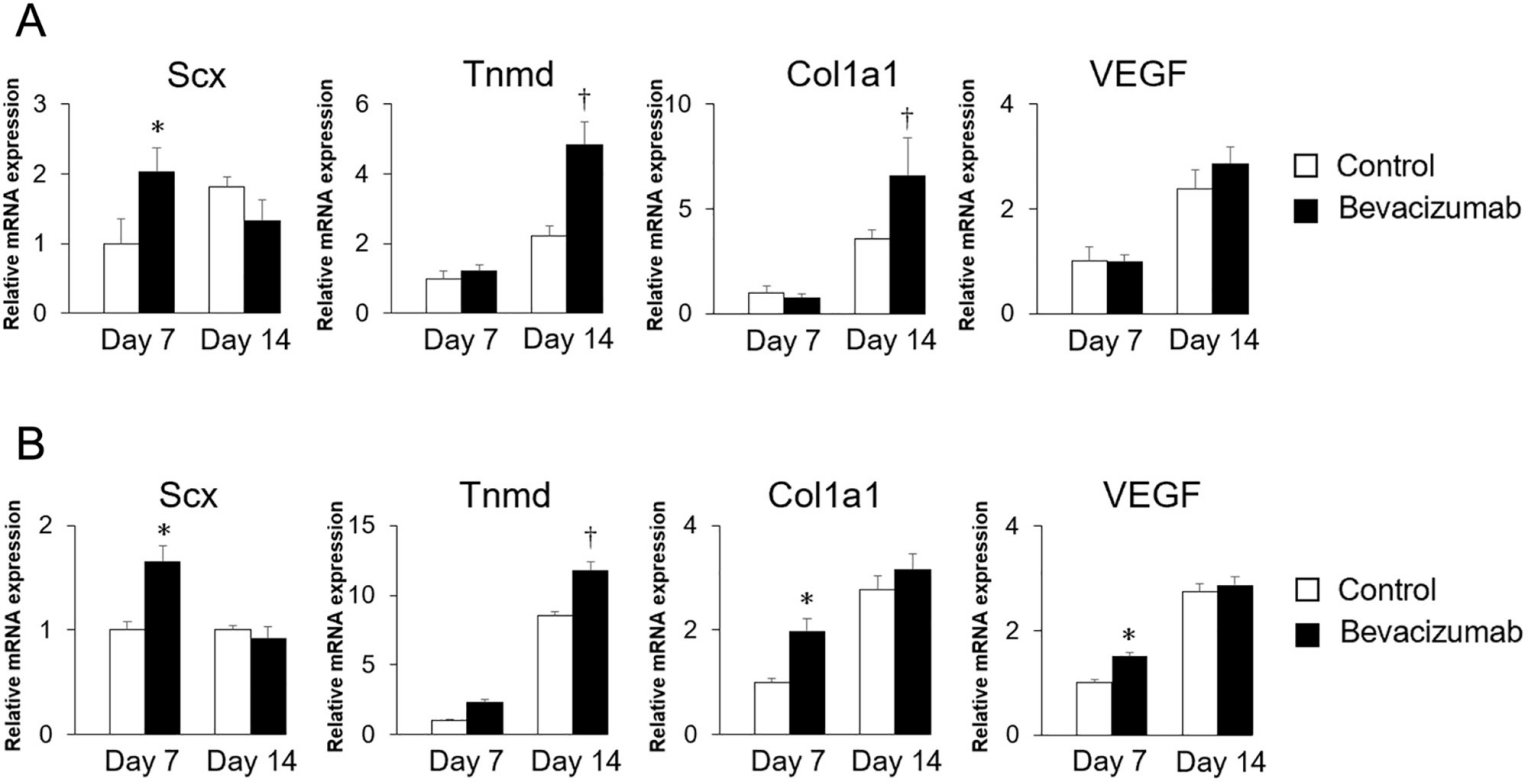
Effects of bevacizumab on the expression of tenogenic differentiation–related genes in TSPCs. PDCs and TDCs were cultured in 3D with 100 μg/mL of bevacizumab for 14 days. Relative mRNA expressions of the *Scx*, *Tnmd*, *Col1a1*, and *VEGF* genes in PDCs (A) and TDCs (B) on days 7 and 14 of culture with bevacizumab versus control cells. N = 5, *P<0.05 vs control on day 7; †*P*<0.05 vs control on day 14.

To assess the effect of bevacizumab on tenogenic differentiation in PDCs, expression of the *Scx*, *Tnmd*, and *Col1a1* genes was analyzed using real-time PCR (Fig 3A). Relative mRNA expression of *Scx* was significantly increased with bevacizumab on day 7 (*P*<0.05), but decreased on day 14. In contrast, control cells showed delayed upregulation of *Scx* on day 14. Relative *Tnmd* mRNA expression was significantly increased with bevacizumab treatment on day 14 (*P*<0.05), but there was no difference on day 7. Similarly, relative *Col1a1* mRNA expression was significantly increased with bevacizumab treatment on day 14 (*P*<0.05), but there was no difference on day 7. No significant difference in relative *VEGF* mRNA expression between bevacizumab-treated cells and control cells was observed on day 7 or day 14 (Fig 3B).

The effect of bevacizumab on tenogenic differentiation was also assessed in TDCs using real-time PCR (Fig 3B). Relative *Scx* mRNA expression was significantly increased with bevacizumab treatment on day 7 (*P*<0.05), but decreased on day 14. In contrast, control cells showed no change between day 7 and day 14. Relative *Tnmd* mRNA expression was significantly increased in both bevacizumab-treated and control cells (*P*<0.05), but bevacizumab-treated cells showed a greater increase than control cells on day 14 (*P*<0.05). Relative *Col1a1* mRNA expression was significantly higher in bevacizumab-treated cells than in control cells on day 7 (*P*<0.05), but the expression level was not different on day 14. Relative *VEGF* mRNA expression was significantly higher in bevacizumab-treated cells than control cells on day 7 (*P*<0.05), but the expression level was not different on day 14 (Fig 3B).

### Immunofluorescent staining of Tnmd

**Fig 4:**
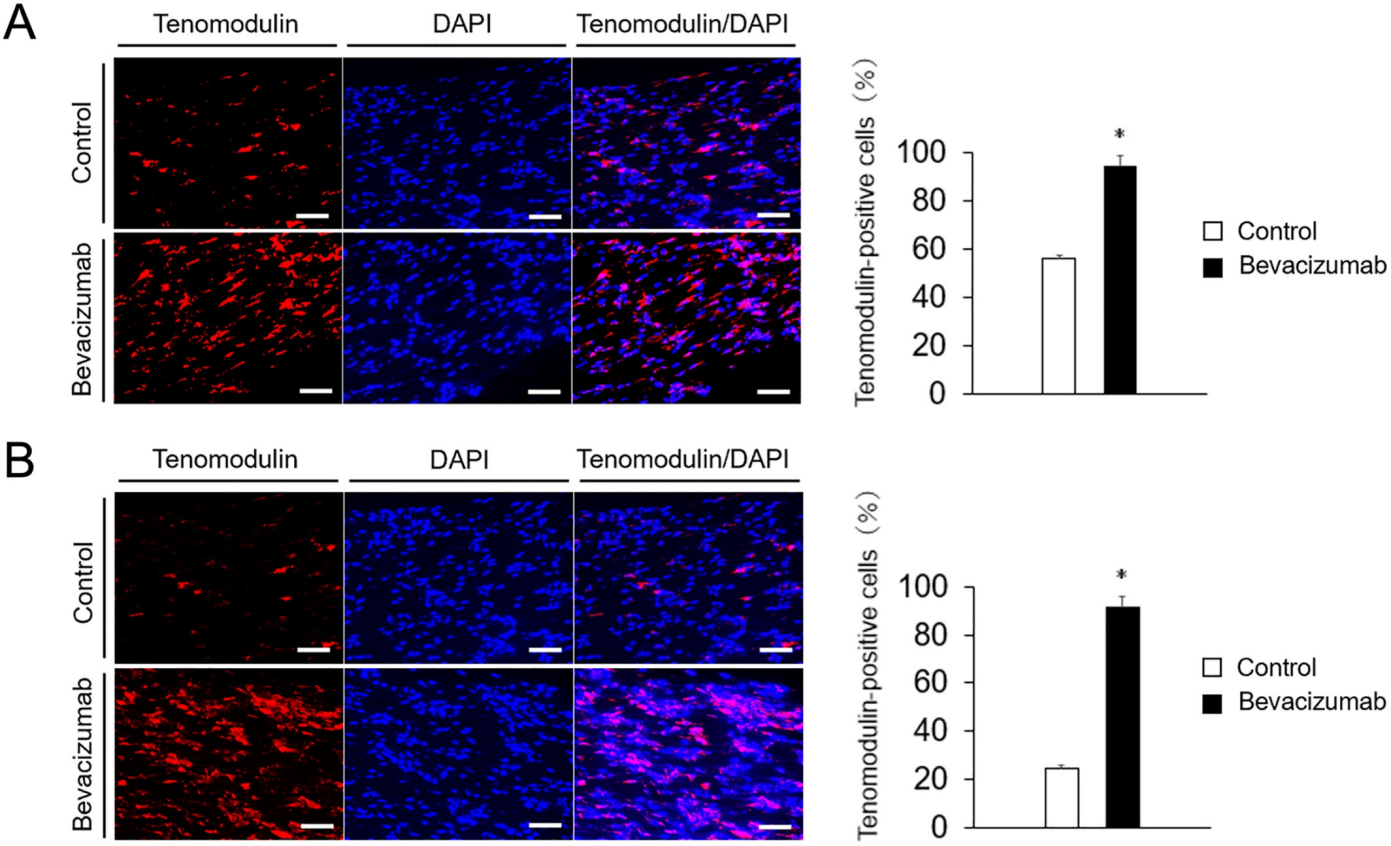
Immunofluorescent staining of Tnmd. PDCs and TDCs were cultured in 3D for 14 days and then immunostained. Using the BZ-X800 analyzer software, the Tnmd-positive cells were quantified relative to DAPI and expressed as a percentage. Immunofluorescence staining of Tnmd in PDCs (A) and TDCs (B) with bevacizumab versus control cells. N = 4, *P<0.05 vs control. Scale bar = 50 μm.

To confirm tenogenic maturation of tendon-like tissue derived from TSPCs, localization of Tnmd expression was investigated based on immunofluorescent findings on day 14. PDCs treated with bevacizumab showed stronger staining of Tnmd than control cells. The percentage of Tnmd-positive cells among PDCs treated with bevacizumab was significantly higher than that of control cells (*P*<0.05) (Fig 4A). TDCs treated with bevacizumab also showed stronger staining of Tnmd than control cells. The percentage of Tnmd-positive cells among TDCs treated with bevacizumab was significantly higher than that of control cells (*P*<0.05) (Fig 4B). The expression of Tnmd increased more noticeably than the mRNA expression levels shown in Fig 3B. This discrepancy in expression levels is attributed to setting a lower threshold for Tnmd positivity on immunostaining. Furthermore, it is well-known that there can be discrepancies between messenger RNA expression levels and protein expression levels.

### Western blotting

**Fig 5:**
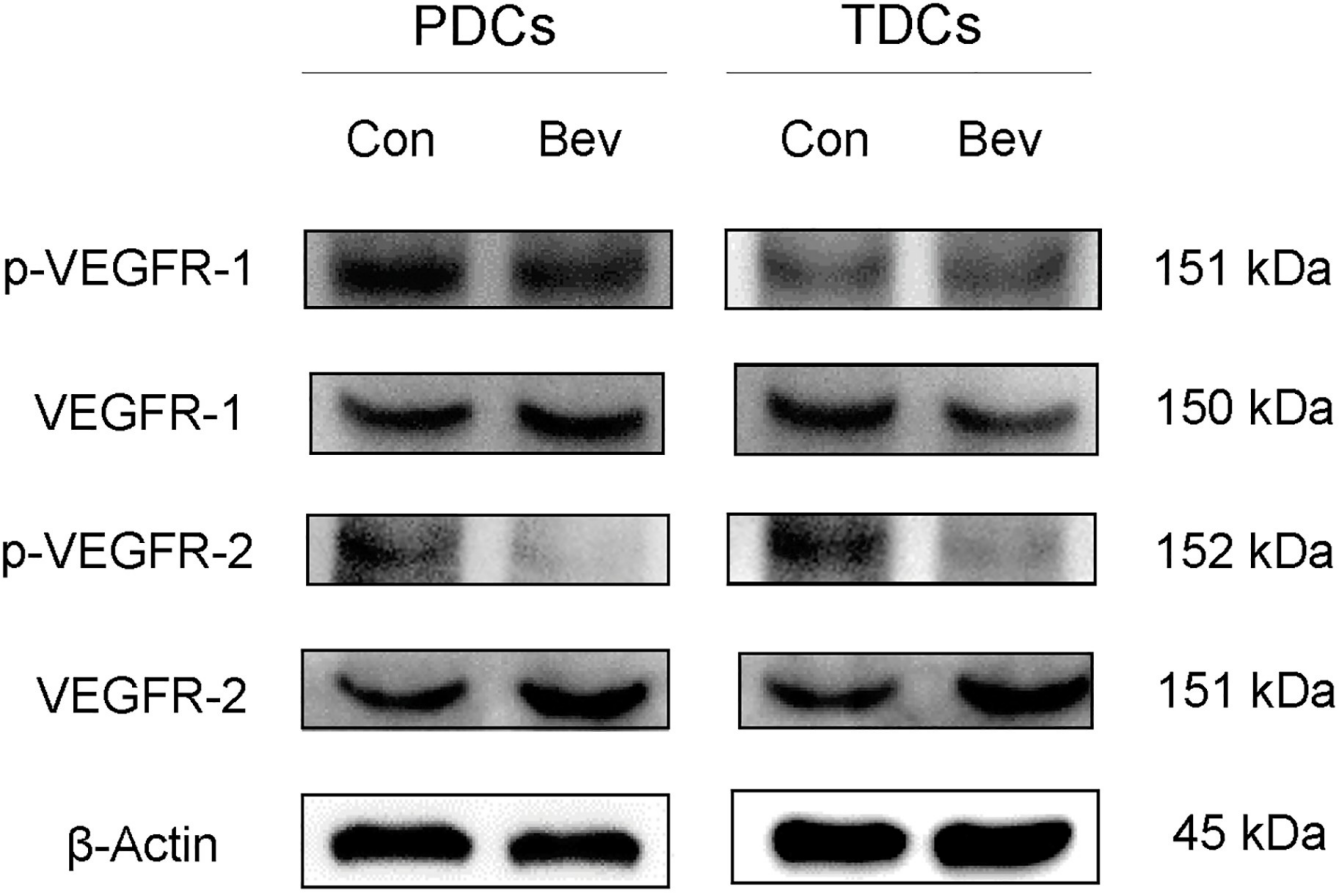
Western blotting to investigate the effects of bevacizumab on VEGF signaling. Western blotting of VEGFR-1, pVEGFR-1, VEGFR-2, and pVEGFR-2 in PDCs and TDCs on day 14 of culture with bevacizumab (Bev) versus control cells (Con).

VEGFR is the receptor for VEGF, and VEGFA binds to VEGFR-1 and VEGFR-2, thereby transmitting signals. To confirm the inhibitory effect of bevacizumab on VEGFR signaling, the expression of phosphorylated VEGFR-1,2 (pVEGFR-1,2) was investigated using western blotting analysis. Bevacizumab did not change the protein expressions of VEGFR-1 and pVEGFR-1 in both PDCs and TDCs compared with control cells. However, though there was no change in the protein expression of VEGFR-2, bevacizumab suppressed the expression of pVEGFR-2 protein in both PDCs and TDCs (Fig 5). Bevacizumab selectively inhibited VEGFR-2, which is more closely associated with the VEGF response in the tendon [9].

### Effects of bevacizumab on proliferation, migration, and toxicity

In the proliferation of PDCs and TDCs, no significant difference was observed among the control, bevacizumab 10 μg/mL, and 100 μg/mL groups (*P*<0.05) (S2 Fig). Similarly, in the migration of PDCs and TDCs, no significant difference was observed among the same groups (*P*<0.05) (S3 Fig). These results were analyzed using the Kruskal-Wallis test.

Apoptosis in PDCs and TDCs cultured for 14 days was examined. In relation to DAPI, the ratio of red in PDCs was on average 7.45% for the control group and 6.45% for the bevacizumab group. In TDCs, the control group exhibited an average of 4.55%, whereas the bevacizumab group showed 5.0%. No significant differences were observed among these values (S4 Fig).

## Discussion

The most important finding of the present study was that bevacizumab promoted *in vitro* tenogenic differentiation and maturation of two distinct TSPCs, TDCs and PDCs, derived from rat Achilles tendon. The effect of bevacizumab was also confirmed by attenuation of the phosphorylation of VEGFR-2.

Bevacizumab is a humanized monoclonal antibody that specifically binds to VEGF-A. This action inhibits the biological activity of VEGF-A, suppressing angiogenesis. Cancer cells require the formation of new blood vessels for growth and proliferation, and bevacizumab is used clinically as an anticancer agent for this reason. However, since angiogenesis is also a normal physiological function, there are concerns that bevacizumab might inhibit the proliferation and migration of normal cells, leading to cytotoxicity. Therefore, bevacizumab was added to TSPCs, and their proliferation and migration were examined using the CCK-8 cell proliferation assay and scratch assay, respectively. Apoptosis was investigated using the TUNEL apoptosis assay. It was found that bevacizumab did not inhibit the proliferation or migration of TSPCs, and there was no increase in apoptosis (S2–S4 Figs).

In this study, the signaling pathways involved in promoting TSPC differentiation when VEGFR-2 is inhibited were not investigated. Various signaling pathways exist; for instance, the JAK-STAT pathway has recently been highlighted for its role in signaling related to TSPC via VEGFR-2. Activation of VEGFR-2 can stimulate the JAK-STAT pathway [10], potentially inhibiting differentiation and proliferation of TSPCs, and it is implicated in tendon cell aging [11]. In this study, by blocking VEGFR-2, it is conceivable that this pathway might have been disrupted, thereby promoting the differentiation of tendon cells. However, this remains a topic for future research.

TSPCs exhibiting self-renewal capability, clonogenicity, and pluripotency were first identified within the tendon fascicle [2], and they are also potentially present in the epitenon/paratenon [4, 12]. These TSPCs express surface markers such as Sca1+, CD90+, CD44+, CD18-, CD34-, CD31-, and CD133-. When cultured in differentiation-inducing media, they exhibit adipogenic, osteogenic, and chondrogenic differentiation, demonstrating their multipotency [8, 13, 14]. Mienaltowski et al. suggested that there is regional distribution of two subpopulations of TSPCs, one originating from the tendon proper and the other from the epitenon/peritenon, and they found that TDCs exhibit greater expression of the tenogenic markers Scx and Tnmd than do PDCs [8, 15]. Although promoting intrinsic repair via TDCs appears to be an ideal approach for natural healing of injured tendons, PDCs are predominantly involved in the early response of the healing process [12]. Thus, one therapeutic strategy for tendon repair may be to augment the tenogenic differentiation and maturation of PDCs as an extrinsic contribution to the initial healing process, and the present study thus focused on promoting tendon-specific differentiation of PDCs by blocking an inhibitory factor.

Tnmd is predominantly expressed in the tendon and ligament, and it is essential for tendon development, maintenance, and healing. Loss of Tnmd reduces the proliferation of tenocytes and the production of collagen fibers in the extracellular matrix [16]. Tnmd overexpression was shown to promote tenogenic differentiation and tendon-like tissue formation in murine mesenchymal stem cells [17]. Tnmd is also involved in the maintenance and function of TSPCs [18]. TSPCs from a *Tnmd*-knockout (*Tnmd*^*−/−*^) mouse line exhibited significantly reduced migration and proliferation potential. In addition, Tnmd is required for the prevention of adipocyte accumulation and fibrovascular scar formation during early tendon healing [19]. Tnmd is a useful phenotypic marker of tenogenic differentiation in terms of maturation and function, and in the present study, it was primarily used to monitor the change in TSPCs. The involvement of TSPCs exhibiting sustainable expression of Tnmd appears to be a key to successful tendon repair.

Scx acts as a transcriptional activator for Tnmd in tenocytes [20]. However, in Fig 3, while the expression of Scx increases on day 7, there is no change in Tnmd expression. On day 14, there’s no change in Scx expression, but Tnmd expression is elevated. To clarify this discrepancy, further experiments are required in the future.

Angiogenesis is a physiological process in wound healing, but uncontrolled vessel growth or impaired vessel regression can lead to scar formation [21]. Although the application of endogenous VEGF has been explored as a means to promote tendon repair [22–24], there are conflicting views regarding the role of VEGF in the healing process [25]. In the tendon healing process, the endogenous VEGF expression level increases in the early stage and decreases in subsequent stages [26–28]. An increased vascular response to exogenous VEGF may contribute to the tendon healing only in the early stage; in contrast, suppression of the vascular response may enhance the healing potential in later stages [29]. The adult tendon is a poorly vascularized tissue, and most of the intrinsic tendon-derived cells are negative for CD34 as an endothelial cell marker [30]. Since hypervascularity occurs in a pathological condition, such as tendinopathy, prolonged angiogenesis with exogenous VEGF in adult tendons can inhibit tendon healing and functional recovery [31]. Thus, controlling angiogenesis may be important for proper tendon repair.

Pre-clinical studies of anti-VEGF therapy for tendon repair using animal models demonstrated effects in reducing angiogenesis and improving tendon collagen organization and mechanical properties [29, 32, 33]. These *in vivo* studies concluded that suppression of angiogenesis plays a role in improvement associated with tendon repair, but no effects on TSPC proliferation, migration, and differentiation were shown. A previous study showed that PRP administration leads to increased expression of the *Scx* and *VEGF* genes in TSPCs, but decreased *Tnmd* expression [7]. The present study showed that VEGF directly reduces the expression of *Tnmd* in TSPCs. Tnmd has anti-angiogenic effects [34], and TSPCs can express Tnmd [2]. Elevated VEGF levels may lead to reduced expression of Tnmd in TSPCs. The present study investigated the direct response of TSPCs to an anti-VEGF agent and confirmed the stimulatory effect on TSPC differentiation and maturation, suggesting an involvement at the cellular level.

The healing process in injured tendons is characterized by three overlapping phases: inflammation, proliferation, and remodeling. Although treatment strategies are required for each phase, the structure and function of the tendon ultimately need to be restored. As described above, VEGF accumulates over time at the repair site and diminishes in later stages of the healing process. PDCs involved in the initial tendon repair process [4] and their tenogenic differentiation and maturation seem to play a key role in successful tendon repair. Since prolonged expression of VEGF in PDCs contributing to tendon repair can inhibit the natural healing process, attenuation of VEGF at appropriate times may stimulate healing and tendon repair. A previous study demonstrated that PRP promotes the migration, proliferation, and tenogenic differentiation of TSPCs via the upregulation of *Scx* expression, but downregulates the expression of *Tnmd* in conjunction with upregulated expression of *VEGF* [7]. Combined administration of PRP and bevacizumab may be a suitable treatment option for enhancing tendon healing.

## Conclusions

This study provides insight into the modulatory effect of bevacizumab on the tenogenic differentiation of two distinct TSPCs, TDCs and PDCs. Since the previous studies demonstrated that both types of TSPCs contribute to tendon repair, the induction of tenogenic differentiation and maturation in these cells appears to be a useful treatment strategy for promoting tendon healing. Attenuating VEGF levels in TSPCs by administration of bevacizumab is a novel candidate therapeutic option for promoting tendon repair.

## Supporting information

S1 Fig Western blot analysis of Tnmd expression in tendon extract. The anti-Tnmd antibody showed a band at 45 kDa in western blotting with PDCs and TDCs cultured for 14 days, confirming its specificity.
(TIF)

S2 Fig Effect of bevacizumab on the proliferation. We used the CCK-8 cell proliferation assay. 24 hours after culturing (A)PDCs and (B)TDCs, Bevacizumab was administered at concentrations of 10μg/ml and 100μg/ml. Evaluations were made on days 1, 2, and 3. Including the control group, no significant difference was observed among the groups. N = 4.
(TIF)

S3 Fig Effect of bevacizumab on the migration. We employed the scratch assay (Oris cell migration assay). After 24 hours of culturing PDCs and TDCs, the stopper was removed, and bevacizumab was administered at concentrations of 10μg/ml and 100μg/ml. After an additional 24 hours, the samples were stained and analyzed using the Keyence BZ-X800. We measured the percentage of the blue area relative to the entire circle area (A). In both PDCs and TDCs, there was no significant difference among the three groups, including the control group (B). N = 3.
(TIF)

S4 Fig Effect of bevacizumab on toxicity. We used the TUNEL apoptosis assay to investigate whether bevacizumab induces apoptosis in PDCs and TDCs cultured for 14 days. In the same way as the tenomodulin immunostaining in our study (Fig 1), we prepared tissue sections and stained them. Images of apoptosis (red) and DAPI (blue) were overlaid, and the percentage of apoptosis-positive cells was determined by BZ-X800. There was no significant difference between the two groups in both PDCs and TDCs. N = 4. White scale bar = 50 μm.
(TIF)

S5 Fig Dose response test for VEGF administration to Tnmd. After 4 days of culturing PDCs and TDCs, VEGF was administered at concentrations of 1ng/ml, 10ng/ml, and 100ng/ml. Three days later, the expression of Tnmd was evaluated using real-time PCR. In both PDCs and TDCs, the groups treated with 10ng/ml and 100ng/ml showed a significant reduction in Tnmd expression compared to the control group. N = 4. *P<0.05.
(TIF)

S6 Fig Expression of Tnmd in 2D-cultured TDCs treated with bevacizumab. Using the same method described in ’Material and Methods: 2D cell culture of TSPCs and treatment with VEGF’, TDCs were treated with bevacizumab at concentrations of 10μg/ml and 100μg/ml. The expression of Tnmd was measured using real-time PCR. Although the sample size (N) was limited to 2, there was a tendency for Tnmd expression not to increase.
(TIF)

S1 Raw images (PDF)
