# Supplementary figures and images for: Bevacizumab promotes tenogenic differentiation and maturation of rat tendon-derived cells in vitro
Source: PLoS One. 2023 Oct 31;18(10):e0293463. doi: 10.1371/journal.pone.0293463 (PMC10617717; doi:10.1371/journal.pone.0293463)

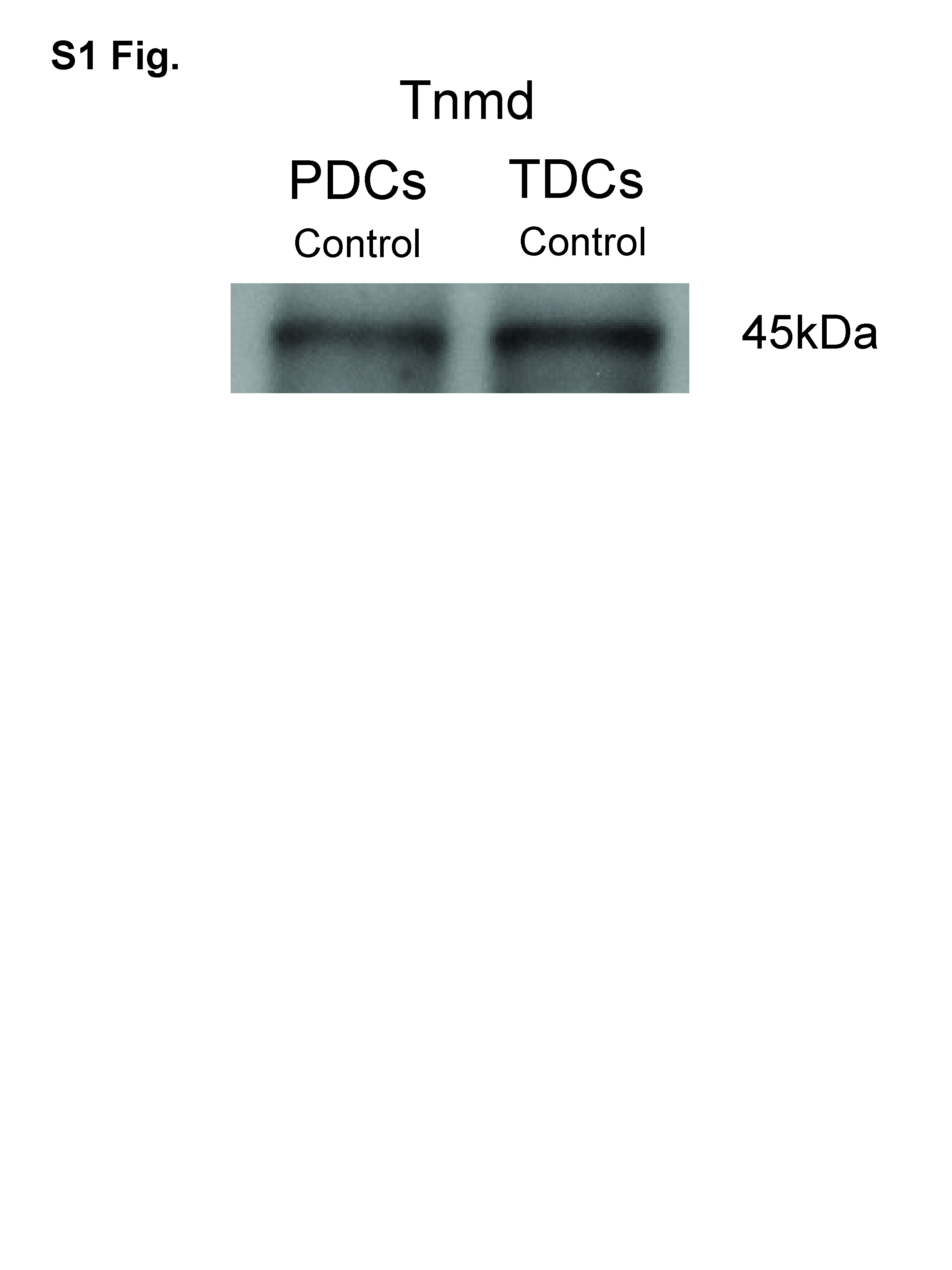

Supplement: S1 Fig — The anti-Tnmd antibody showed a band at 45 kDa in western blotting with PDCs and TDCs cultured for 14 days, confirming its specificity. (TIF) [file pone.0293463.s001.tif]

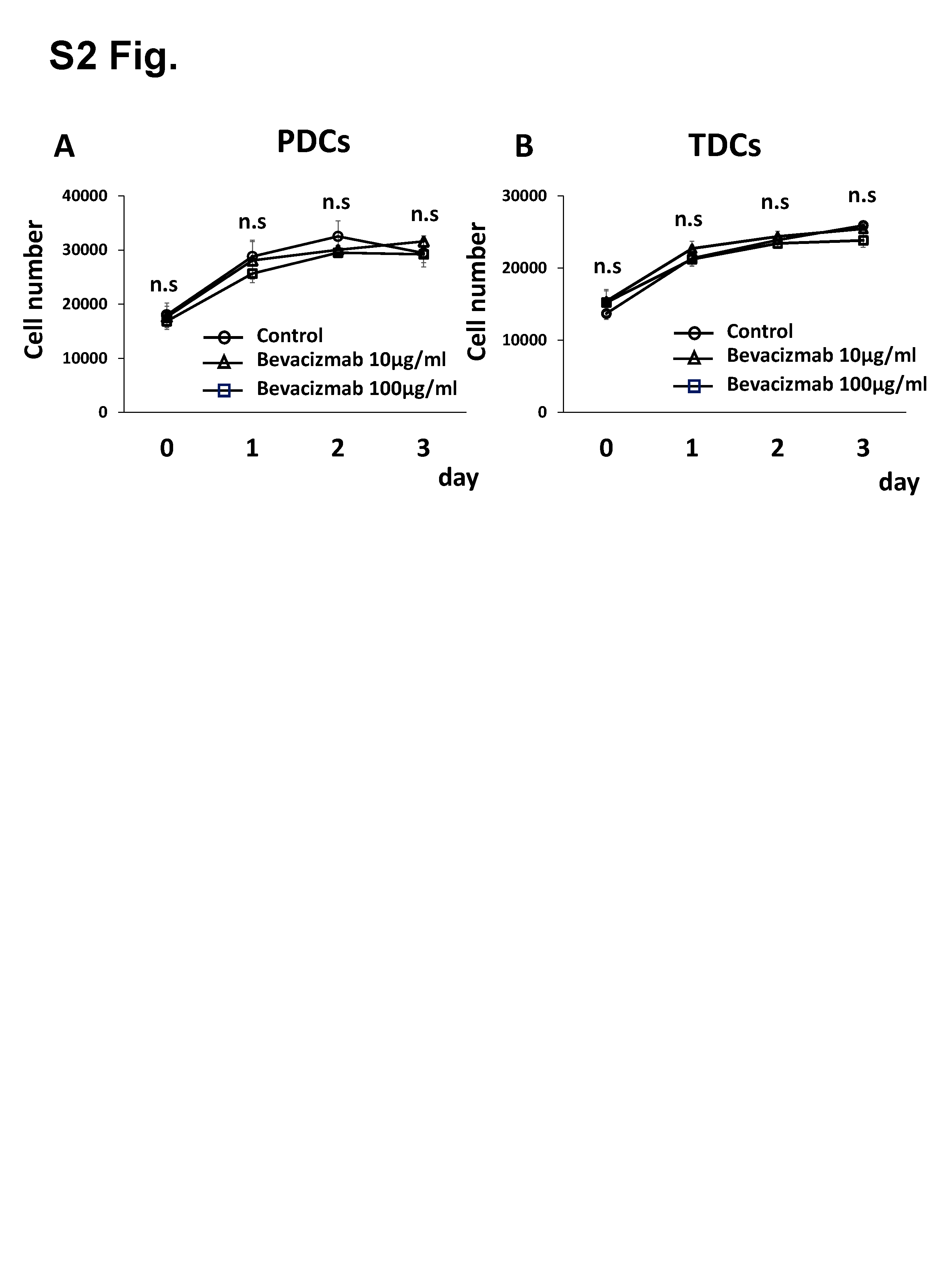

Supplement: S2 Fig — We used the CCK-8 cell proliferation assay. 24 hours after culturing (A)PDCs and (B)TDCs, Bevacizumab was administered at concentrations of 10μg/ml and 100μg/ml. Evaluations were made on days 1, 2, and 3. Including the control group, no significant difference was observed among the groups. N = 4. (TIF) [file pone.0293463.s002.tif]

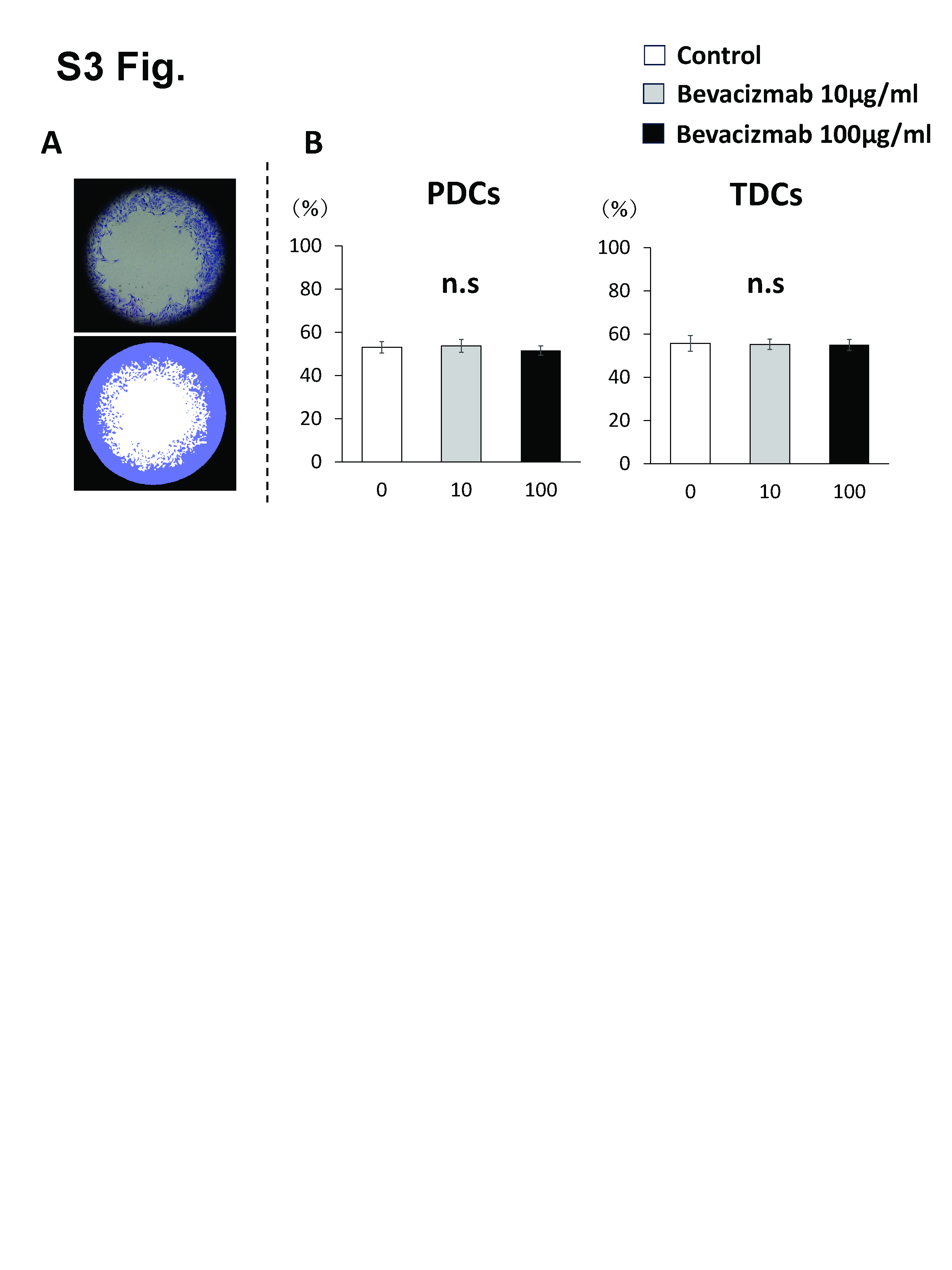

Supplement: S3 Fig — We employed the scratch assay (Oris cell migration assay). After 24 hours of culturing PDCs and TDCs, the stopper was removed, and bevacizumab was administered at concentrations of 10μg/ml and 100μg/ml. After an additional 24 hours, the samples were stained and analyzed using the Keyence BZ-X800. We measured the percentage of the blue area relative to the entire circle area (A). In both PDCs and TDCs, there was no significant difference among the three groups, including the control group (B). N = 3. (TIF) [file pone.0293463.s003.tif]

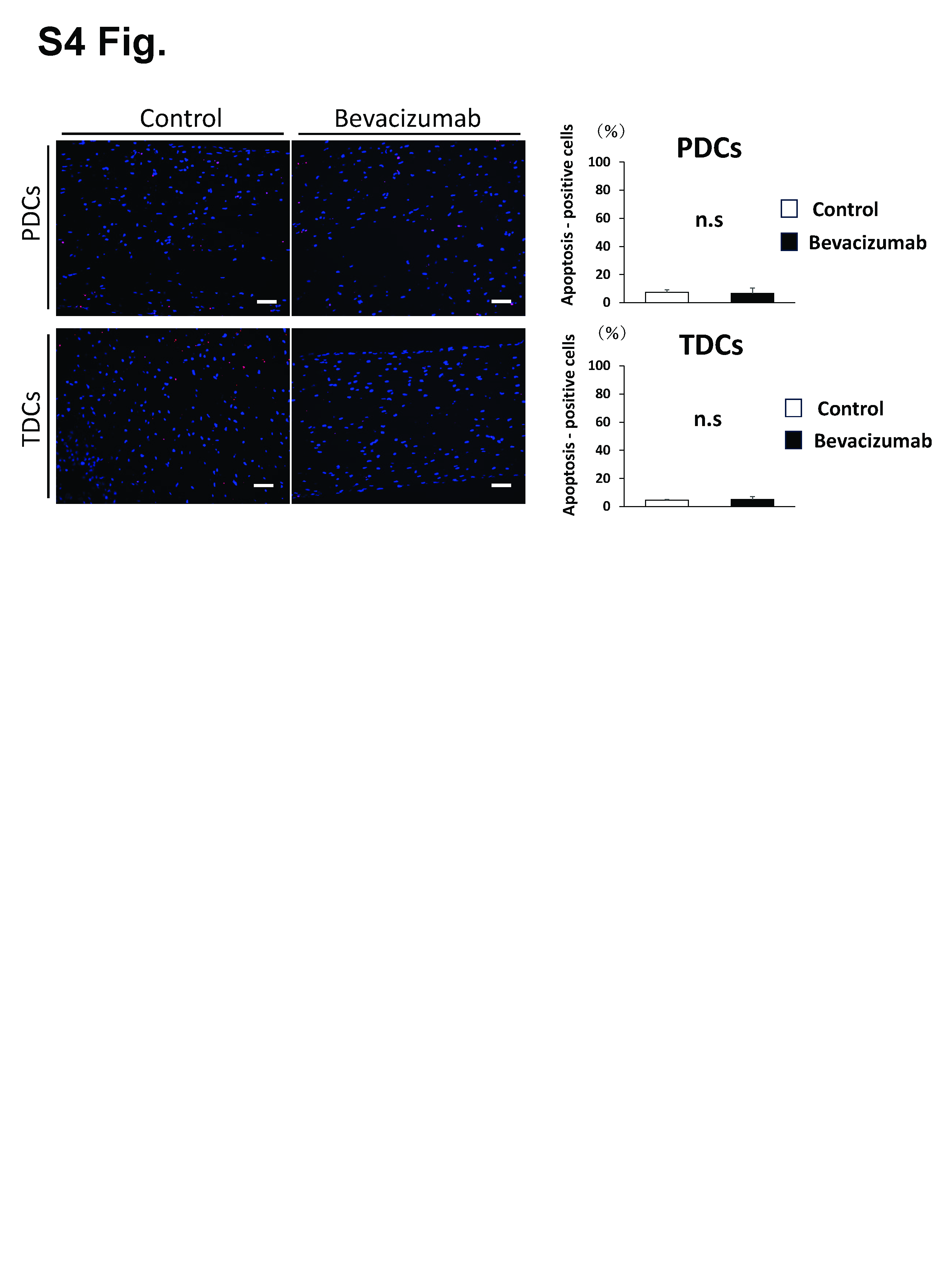

Supplement: S4 Fig — We used the TUNEL apoptosis assay to investigate whether bevacizumab induces apoptosis in PDCs and TDCs cultured for 14 days. In the same way as the tenomodulin immunostaining in our study (Fig 1), we prepared tissue sections and stained them. Images of apoptosis (red) and DAPI (blue) were overlaid, and the percentage of apoptosis-positive cells was determined by BZ-X800. There was no significant difference between the two groups in both PDCs and TDCs. N = 4. White scale bar = 50 μm. (TIF) [file pone.0293463.s004.tif]

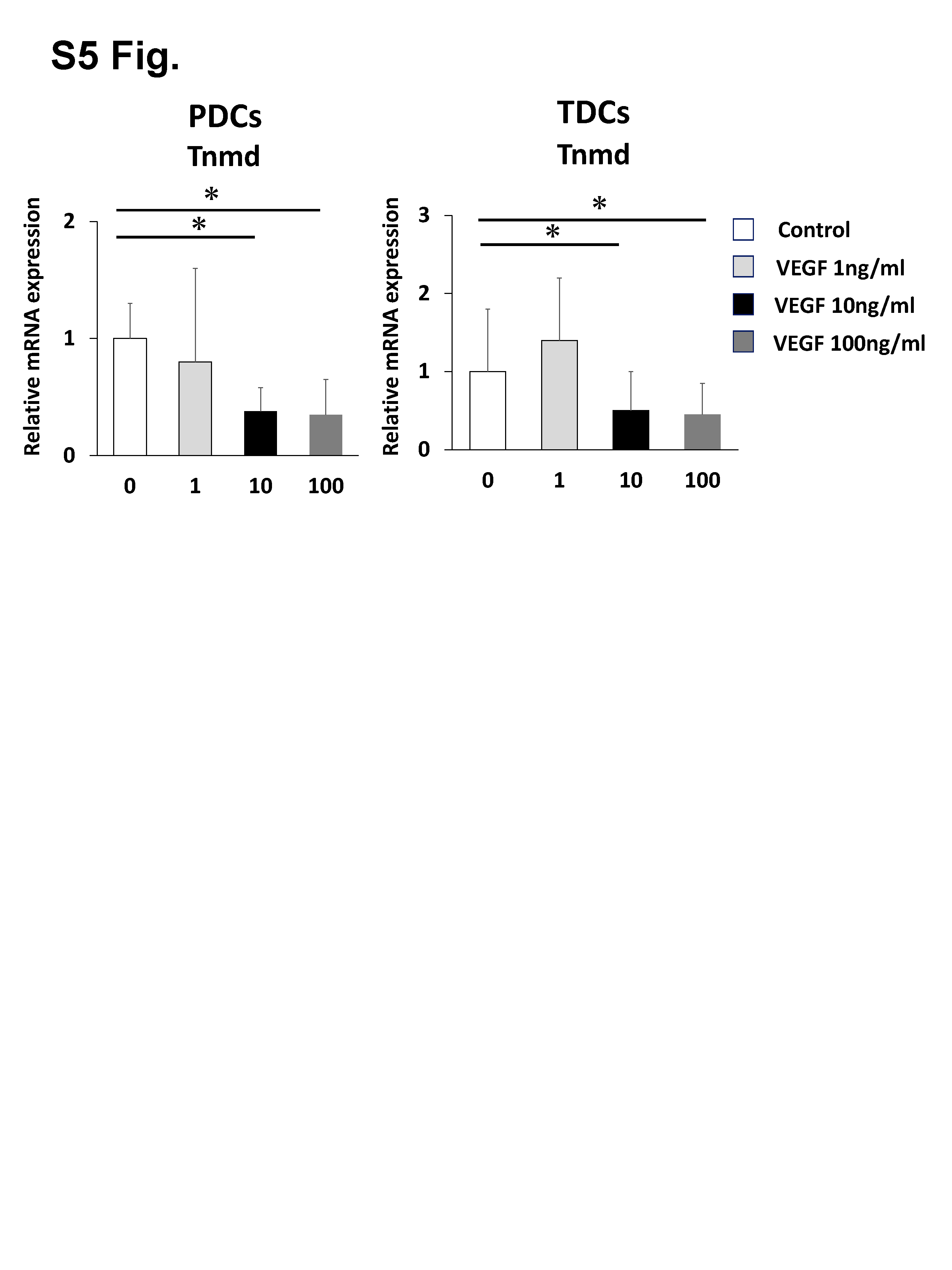

Supplement: S5 Fig — After 4 days of culturing PDCs and TDCs, VEGF was administered at concentrations of 1ng/ml, 10ng/ml, and 100ng/ml. Three days later, the expression of Tnmd was evaluated using real-time PCR. In both PDCs and TDCs, the groups treated with 10ng/ml and 100ng/ml showed a significant reduction in Tnmd expression compared to the control group. N = 4. *P<0.05. (TIF) [file pone.0293463.s005.tif]

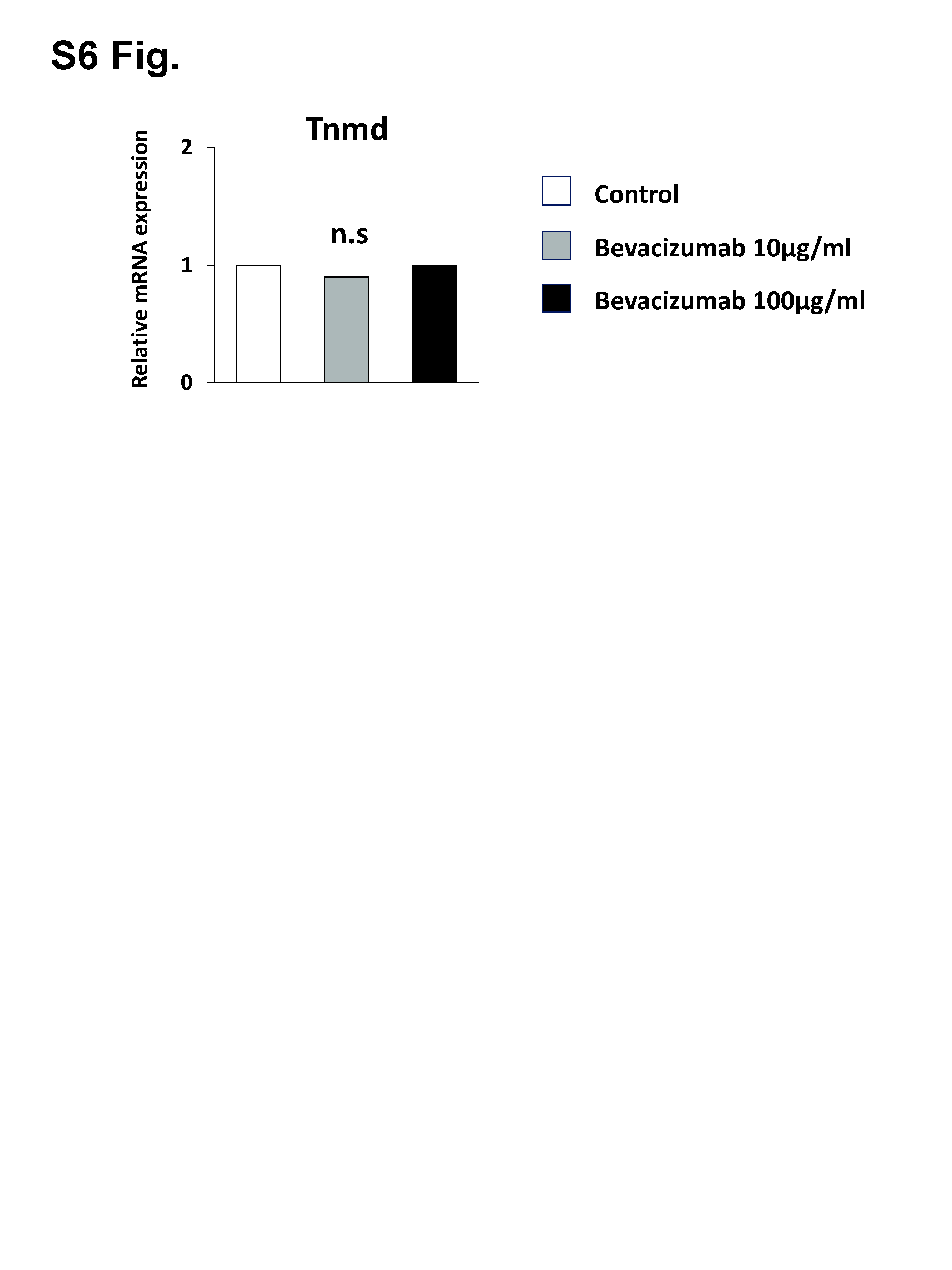

Supplement: S6 Fig — Using the same method described in ’Material and Methods: 2D cell culture of TSPCs and treatment with VEGF’, TDCs were treated with bevacizumab at concentrations of 10μg/ml and 100μg/ml. The expression of Tnmd was measured using real-time PCR. Although the sample size (N) was limited to 2, there was a tendency for Tnmd expression not to increase. (TIF) [file pone.0293463.s006.tif]
