# Supplementary material for: Bevacizumab promotes tenogenic differentiation and maturation of rat tendon-derived cells in vitro
Source: PLoS One. 2023 Oct 31;18(10):e0293463. doi: 10.1371/journal.pone.0293463 (PMC10617717; doi:10.1371/journal.pone.0293463)

Figure 5 raw blots

p-VEGFR-1

PDCs

Con Bev X X

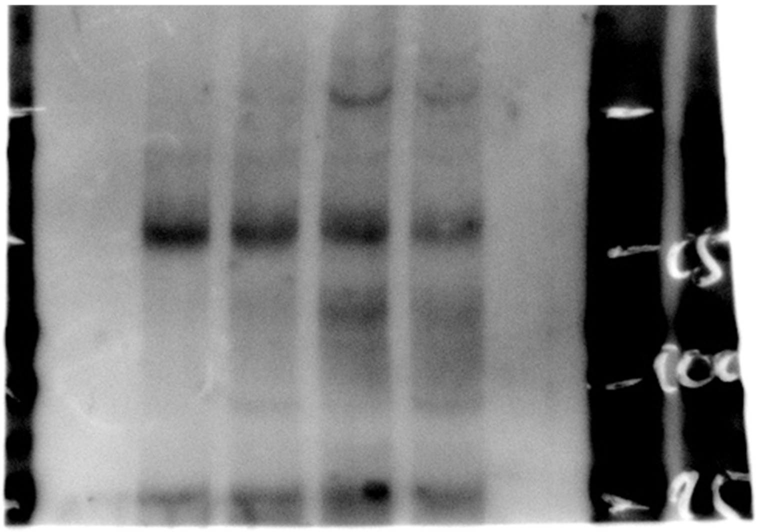

TDCs

X X Con Bev

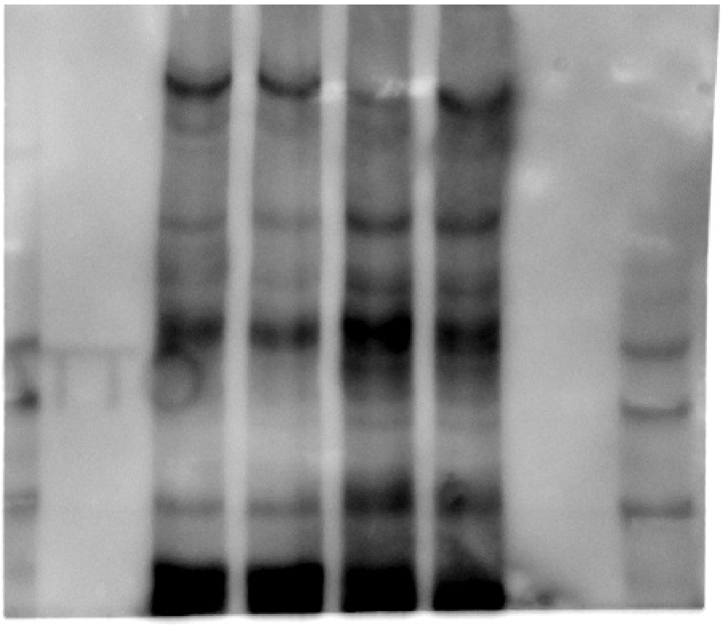

VEGFR-1

	PDCs		TDCs	
	Con	Bev	Con	Bev

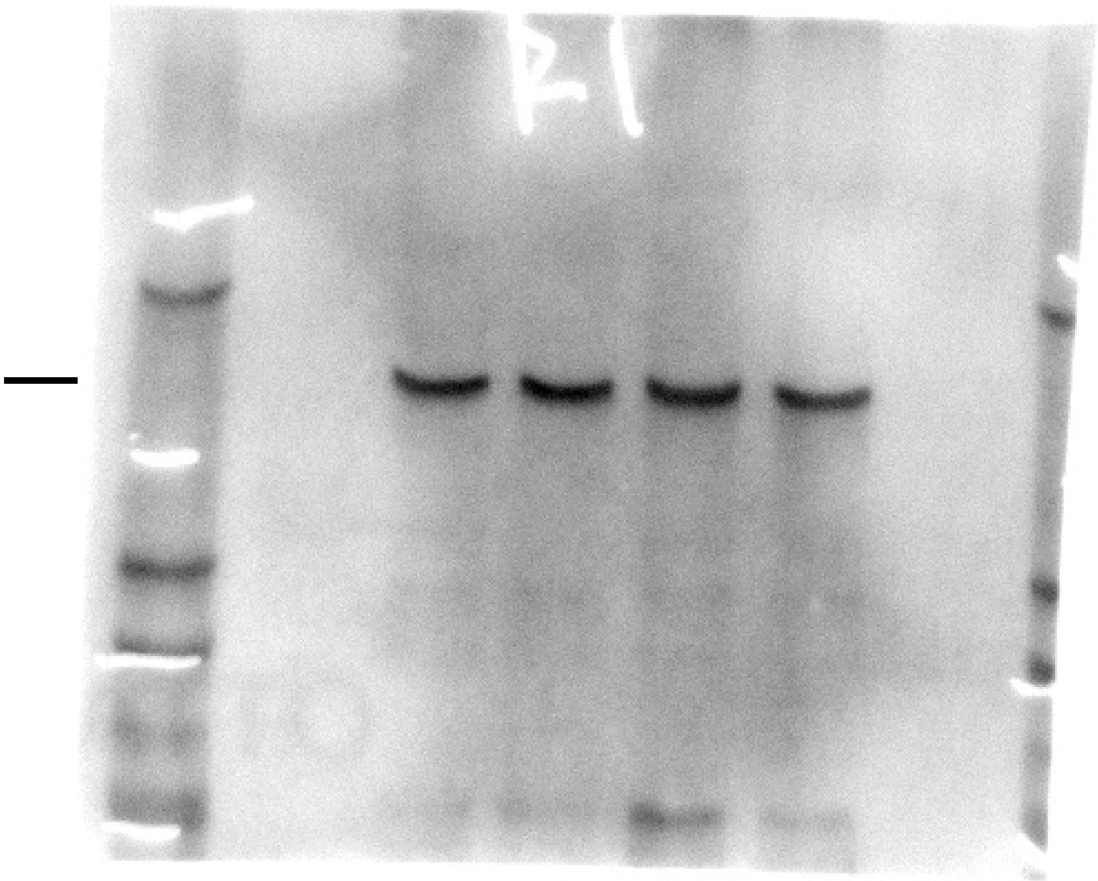

p-VEGFR-2

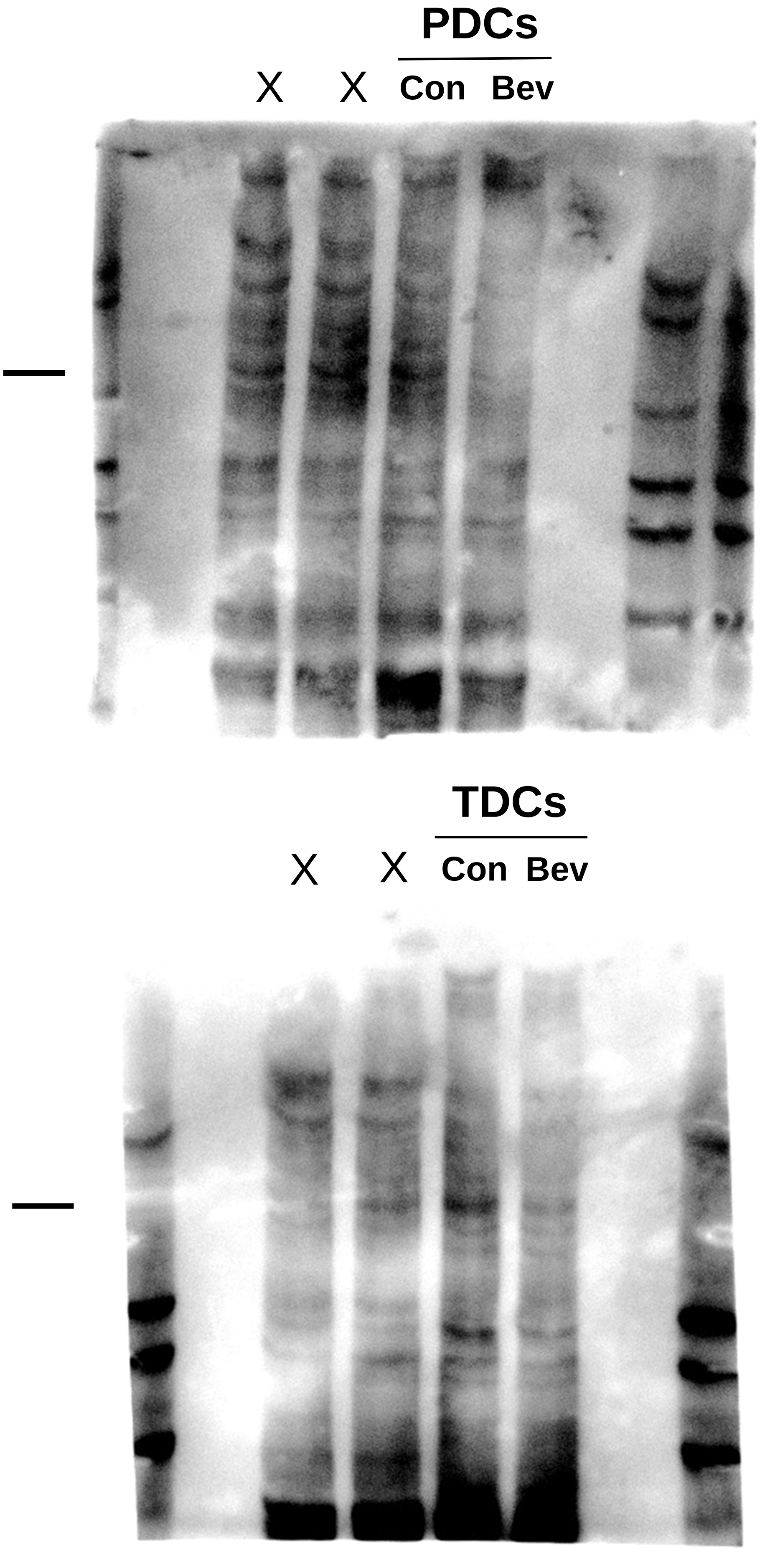

VEGFR-2

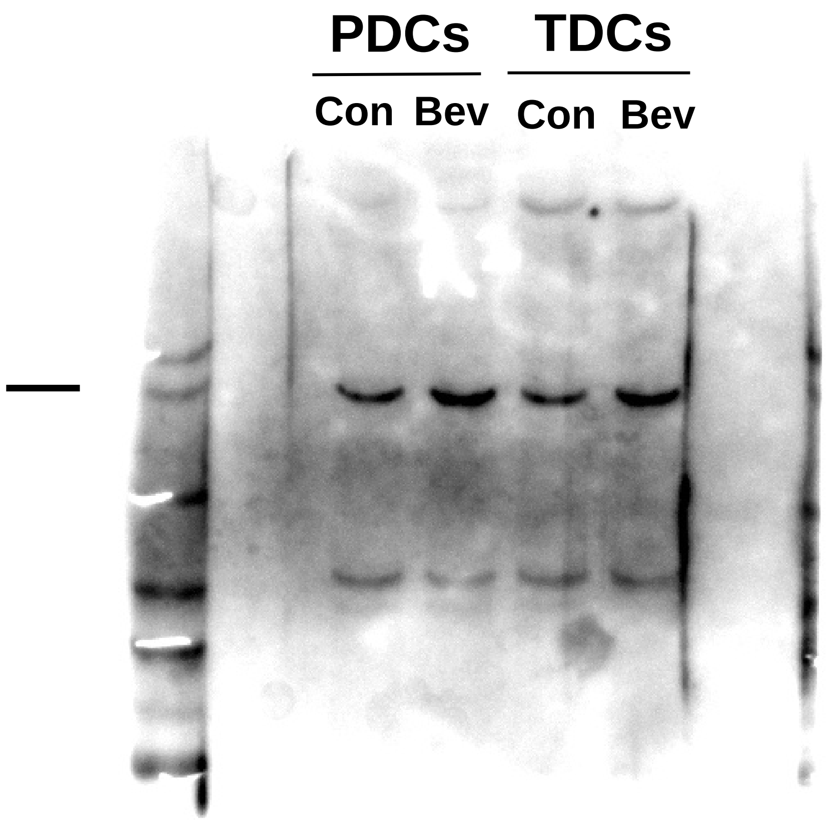

β-Actin

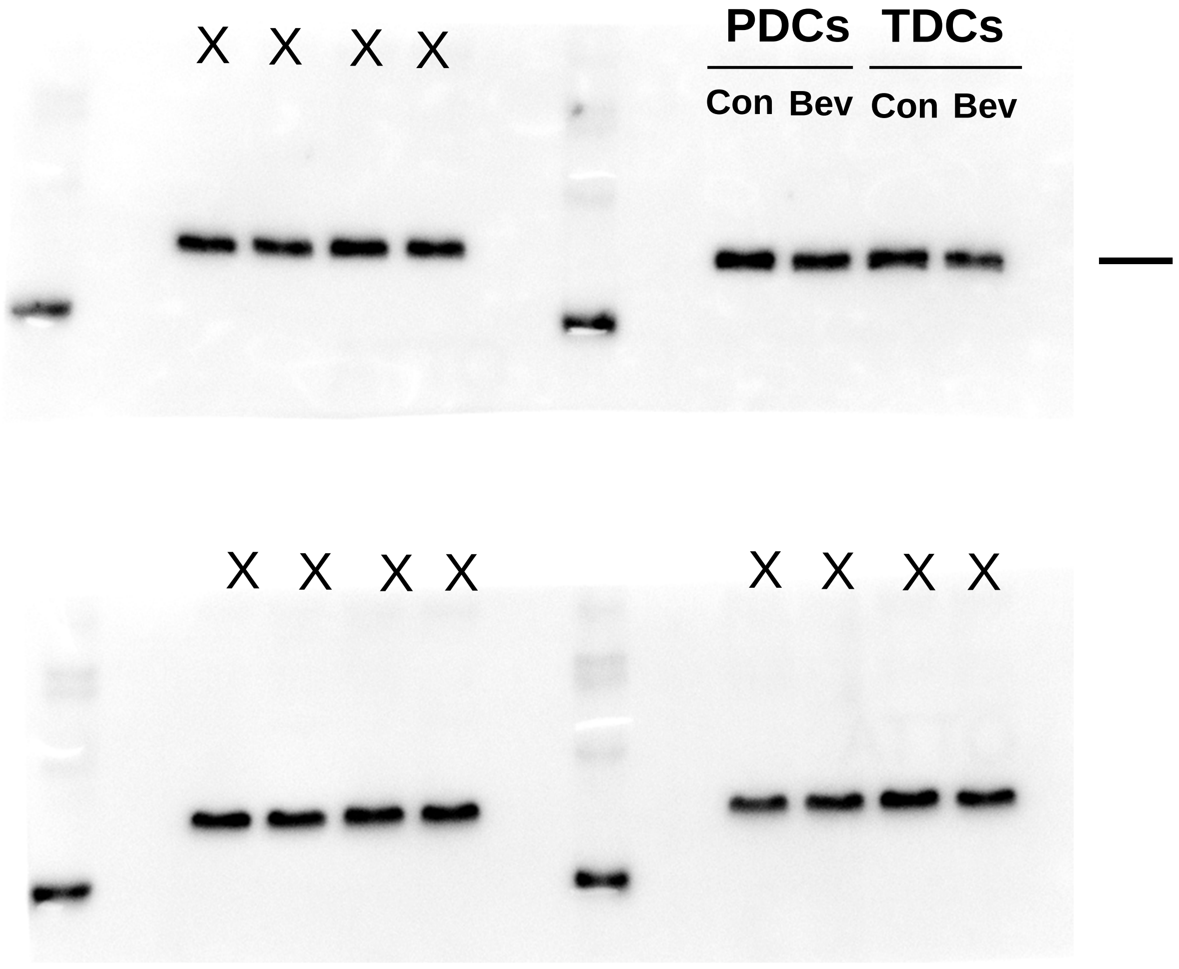

Supplementary 1 Figure raw blots

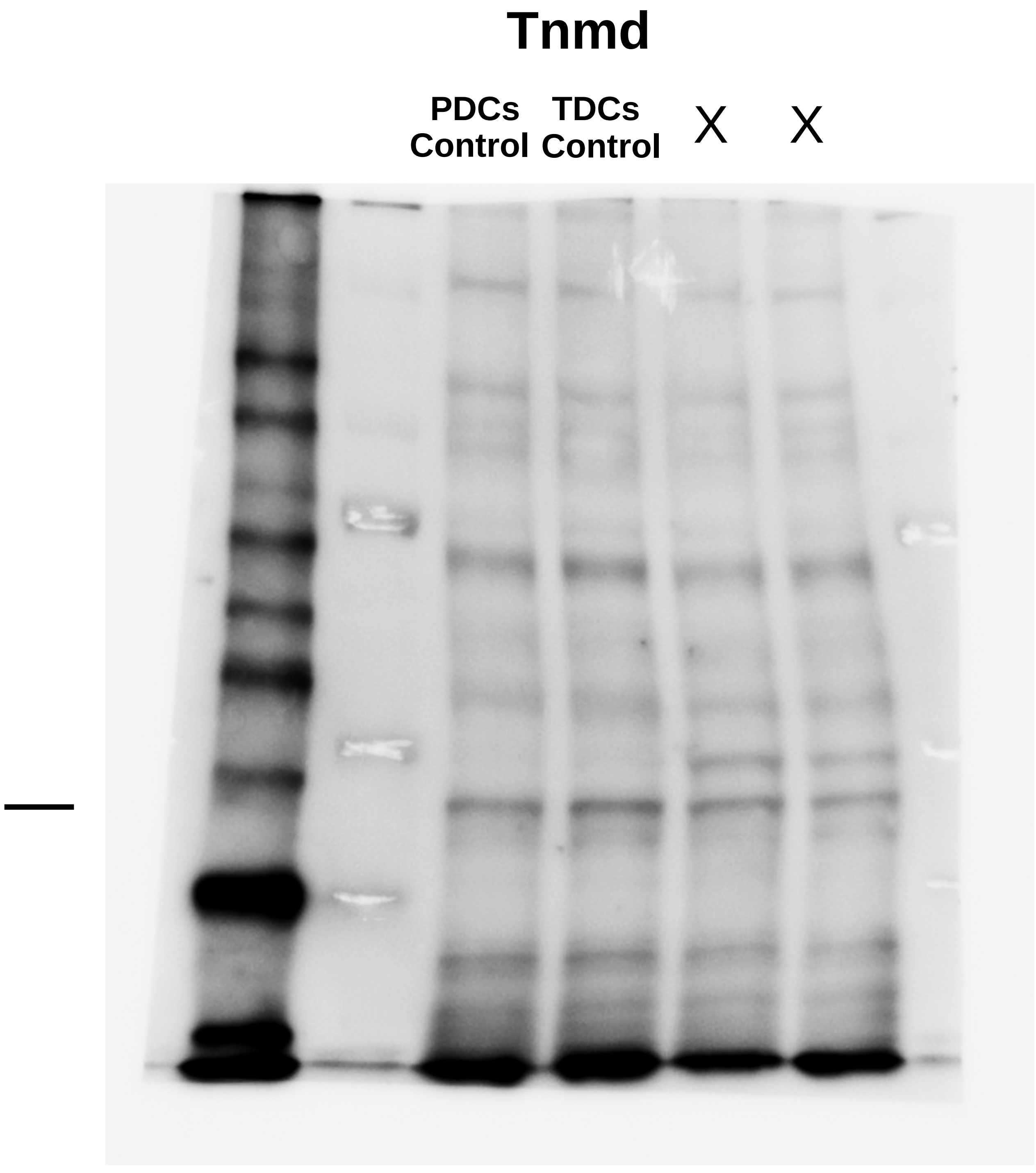

Supplement: S1 Raw images — (PDF) [file pone.0293463.s007.pdf]
